# Phenylacetylglutamine exacerbates sepsis-induced cardiac dysfunction and left ventricular remodeling via the ferroptosis and TLR4/NF-κB pathway

**DOI:** 10.3389/fcvm.2026.1870510

**Published:** 2026-08-13

**Authors:** Xin Dong, Lianhua Yan, Pan Lu, Yang Gong, Guangji Wang

**Affiliations:** 1 Department of Cardiology, The Central Hospital of Wuhan, Tongji Medical College, Huazhong University of Science and Technology, Wuhan, China; 2 Department of Cardiology, Renmin Hospital of Wuhan University, Wuhan, Hubei, China; 3 Cardiovascular Research Institute of Wuhan University, Wuhan, China; 4 Hubei Key Laboratory of Cardiology, Wuhan, China

**Keywords:** electrical remodeling, ferroptosis, phenylacetylglutamine, sepsis, ventricular arrhythmias

## Abstract

**Background:**

Cardiac dysfunction and arrhythmias are serious complications of sepsis-induced cardiomyopathy and are closely associated with increased mortality. The gut microbial metabolite phenylacetylglutamine (PAGln) has been shown to be closely associated with the prognosis of patients with heart failure. However, it remains unclear whether PAGln plays a role in regulating sepsis-induced cardiac dysfunction and ventricular arrhythmias (VAs).

**Objective and methods:**

The objective of this study is to evaluate the effects of PAGln on ventricular remodeling and VAs in mice with sepsis. In this study, we administered intraperitoneal injections of PAGln to mice for 14 days as a pretreatment, followed by stimulation with lipopolysaccharide (LPS) to establish a sepsis model, which was then evaluated using histopathology, molecular biology, cardiac electrophysiology, and echocardiography.

**Results:**

We found that PAGln pretreatment exacerbated LPS-induced cardiac dysfunction and LPS-mediated inflammatory responses. Furthermore, PAGln exacerbated cardiac electrical remodeling and increased susceptibility to VAs in LPS-treated mice. Mechanistically, PAGln may participate in mediating sepsis-induced inflammation, cardiac dysfunction, and susceptibility to VAs by regulating the ferroptosis and TLR4/NF-κB signaling pathway.

**Conclusions:**

This study preliminarily demonstrates that the gut microbial metabolite PAGln exacerbates LPS-induced cardiac dysfunction and inflammation and increases susceptibility to VAs, a mechanism that may be associated with the activation of ferroptosis and the TLR4/NF-κB signaling pathway.

## 1 Introduction

Sepsis refers to a systemic inflammatory reaction induced by infection, and severe cases may progress to organ damage and multiple organ failure (1). The inflammatory reaction activated by sepsis secretes plenty of chemokines, interleukins and other inflammatory factors, causing cardiomyocyte injury and cardiac dysfunction (2). Sepsis-induced cardiomyopathy (SIC) refers to cardiac dysfunction caused by sepsis, often accompanied by malignant ventricular arrhythmias (VAs) and a significantly increased risk of cardiac arrest (3, 4). This condition represents a life-threatening emergency that typically deteriorates rapidly within a short timeframe. However, the pathogenesis of SIC remains incompletely elucidated, posing a significant challenge to the development of effective therapeutic strategies targeting SIC.

The gut microbiota, functioning as an endocrine organ within the human intestine, participates in the metabolism of the organism and influences the pathophysiology of various diseases. Research has revealed that sepsis induces severe dysbiosis, while the gut microbiota can directly or indirectly affect the pathophysiological processes of the host's intestinal and extraintestinal organs through bioactive metabolites (5, 6). In recent years, multiple studies have demonstrated that targeted modulation of gut microbiota metabolites plays a crucial role in disease progression. For instance, indole-3-propionic acid can mitigate infection by enhancing macrophage phagocytosis, thereby preventing sepsis (7). In addition, the gut microbiota-derived metabolite Proline-Leucine dipeptide exacerbates sepsis-induced acute lung injury by activating the NOD2/NF-κB signaling pathway (8).

Phenylacetylglutamine (PAGln) is a microbial metabolite derived almost entirely from bacterial phenylalanine metabolism, formed by the combination of phenylacetic acid and glutamine (9). Evidence indicates that circulating PAGln levels exhibit a strong clinical association with heart failure and major adverse cardiovascular events (10–12). Furthermore, recent studies indicate PAGln participates in regulating cardiac remodeling and susceptibility to arrhythmias (13, 14). However, the impact of PAGln on sepsis-induced cardiomyopathy remains unclear. This study aims to investigate the effects of PAGln on sepsis-induced cardiac dysfunction and VAs susceptibility, and explore its underlying mechanisms.

## 2 Materials and methods

### 2.1 Animals and treatments

All experimental procedures involving animals adhered to the principles stated in the 2011 revised edition of the NIH Guide for the Care and Use of Laboratory Animals and approved from the Animal Ethics Committee (Approval No. 2024-07-15B). 8 weeks old male C57BL/6 mice were purchased from the Hubei Provincial Center for Disease Control and Prevention. All experimental mice were housed in an SPF environment, where temperature and humidity were maintained at constant levels, and a 12-hour light/12-hour dark cycle was observed, with free access to food and water throughout the experiment. Sterile saline was used to dissolve lipopolysaccharide (LPS, Sigma) at a dose of 10 mg/kg. Mice received this agent via a single intraperitoneal injection to induce sepsis and subsequent cardiac dysfunction (15). Prior to LPS administration, mice received daily intraperitoneal injections of either the solvent or PAGln (50 mg/kg, Sigma, 28047-15-6) for 14 consecutive days—a dosage selected based on previous literature (12, 16). The mice were divided into four groups: Control, PAGln, LPS, and LPS + PAGln. Experimental animals were individually marked with a bitter acid solution (yellow) to distinguish between different experimental groups. Samples were collected 24 h after LPS injection, all animals were anesthetized via intraperitoneal injection with sodium pentobarbital (50 mg/kg) and subsequently euthanized. Euthanasia was performed by cervical dislocation, in full compliance with the regulations of the Animal Ethics Committee, to minimize animal suffering.

All samples were assigned random numerical codes to replace the original group information, and all identifiable information—such as grouping, treatment, and animal identification numbers—was removed from the labels. The experimental personnel and the personnel evaluating the results were kept separate, and the evaluators were unable to determine the grouping based on the appearance of the samples or the labels.

### 2.2 Biochemical analysis

Serum concentrations of creatine kinase-mb (CK-MB), lactate dehydrogenase (LDH), and aspartate aminotransferase (AST) were measured in the serum of mice from each group using an automated biochemical analyzer (Chemray 800).

### 2.3 Detection of MDA and iron

The Malondialdehyde (MDA) in serum and cardiac were measured by malondialdehyde assay kit. The iron concentration levels in serum and cardiac were measured by an iron assay kit. All operations were carried out in accordance with the protocol of the kits.

### 2.4 Echocardiography

Mice underwent echocardiography examination (China Vinno Technology) 24 h after LPS injection under anesthesia with 1.5%–2% isoflurane. Parameters measured included left ventricular ejection fraction (LVEF), left ventricular fractional shortening (LVFS). The procedures were performed in accordance with previously published literature (17).

### 2.5 Histopathological analysis

Histopathological methods were performed as described in previous literature (17, 18). From each group, ventricular tissue samples from 6 mice were fixed in formalin solution for 24 h, then embedded in paraffin. Subsequently, 3–5 consecutive sections (5μm thick) were prepared. Hematoxylin-eosin (HE) staining was performed for histopathological examination, followed by microscopic observation under an optical microscope. Ventricular tissue sections were subjected to immunofluorescence and immunohistochemical staining using anti-CD68 (Servicebio-GB113109), anti-CX43 (Servicebio-GB11234), anti-4-HNE (Biosss-6313R), anti-GPX4 (proteintech-30,388) antibodies to detect expression levels. Five non-overlapping fields of view were randomly selected from each section and photographed. All images were blindly quantitatively analyzed by two independent researchers under double-blind conditions using Image-Pro Plus software.

### 2.6 Electrophysiological studies

Electrophysiological studies were performed according to the previously reported method (19). Electrophysiological studies conducted in isolated perfused hearts utilized a Langendorff apparatus with HEPES-buffered Tyrode's solution (130 mM NaCl; 5.4 mM KCl; 1.8 mM CaCl₂; 1 mM MgCl₂; 0.3 mM Na₂HPO₄; 10 mM HEPES; 10 mM glucose; pH adjusted to 7.4 with NaOH). The hearts were perfused at 37 °C under constant pressure (60 mmHg) with a 95% oxygen−5% carbon dioxide mixture and evaluated according to a previously established protocol. The perfused hearts were stimulated via a pair of electrodes placed in the right atrium. All isolated hearts were stabilized for 20 min under constant perfusion flow prior to programmed electrical stimulation. S1-S1 stimulation was used to evaluate 90% action potential duration (APD) and the APD alternans threshold (ALT). The pacing cycle length (PCL) was gradually reduced from 150 ms to 30 ms in steps of 5 ms. Each PCL was maintained for 30 s, followed by a 30-second recovery interval to eliminate pacing memory. APD90 was determined as the average 90% repolarization time measured from 6 to 8 consecutive monophasic action potentials (MAPs) at a PCL of 150 ms. APD alternans was identified when the difference in APD90 between two successive beats reached ≥5% for 10 consecutive beats. The ALT was defined as the longest PCL capable of inducing this response. Burst pacing (50 Hz, 2 ms pulse width, 2 s duration) was performed to assess the inducibility of VAs. VAs was defined as **≥**2 s of continuous ventricular ectopic activity.

### 2.7 Quantitative real-time PCR

The qRT-PCR method performed according to previous literature (18). In brief, total RNA was purified from ventricular samples using RNAiso Plus reagent (Takara, Japan). RNA was reverse transcribed into cDNA using the PrimeScript RT Kit (TaKaRa). Subsequently, qRT-PCR was performed in a 20μl reaction system containing cDNA, forward primer, reverse primer, and SYBR Premix Ex Taq® (Takara). The primer sequences used for qRT-PCR are as follows (GAPDH: CATCACTGCCACCCAGAAGACTG, ATGCCAGTGAGCTTCCCGTTCAG; TNF-α: TGATCCGCGACGTGGAA, ACCGCCTGGAGTTCTGGAA; IL-1β: GCAACTGTTCCTGAACTCAACT, ATCTTTGGGGTCCGTCAACT; IL-6: GAGGATACCACTCCCAACAGACC, AAGTGCATCATCGTTGTTCATACA)

### 2.8 Western blot

The Western blot method performed according to previous literature (17). Briefly, total protein was extracted from frozen left ventricular tissue, then protein concentration was measured using the BCA assay kit (Servicebio, Wuhan) and standardized. Proteins were separated by SDS-PAGE and transferred to PVDF membranes. Membranes were blocked with 5% skim milk powder for 1 h, then incubated overnight at 4 °C with different primary antibodies (TNF-α-CST-11948, IL-1β-abcam-ab9722, IL-6-proteintech-21865-1, CX43-Servicebio GB11234, GPX4-proteintech−30388, TLR4-abcam-ab13556, P65-CST-6396, *p*-P65-proteintech-82335-1). Finally, membranes were incubated with secondary antibodies at room temperature for 60 min. Signal detection was performed using the ultra-sensitive ECL kit (Biosharp, Anhui). Protein bands were visualized using a chemiluminescent imaging system.

### 2.9 Statistical analysis

Data were analyzed using GraphPad Prism software (GraphPad Software, San Diego). Continuous variables and categorical variables were expressed as mean ± standard error of the mean (SEM) and number (percentage), respectively. Normally distributed continuous data were analyzed using Student's t-test, while non-normally distributed continuous data were analyzed using the Mann–Whitney U test. Normality was assessed using the Shapiro–Wilk test. Categorical data comparisons were performed using the chi-square test. A two-tailed *P* value < 0.05 was considered statistically significant.

## 3 Results

### 3.1 PAGln exacerbates LPS-induced cardiac dysfunction and myocardial injury in mice

**Figure 1:**
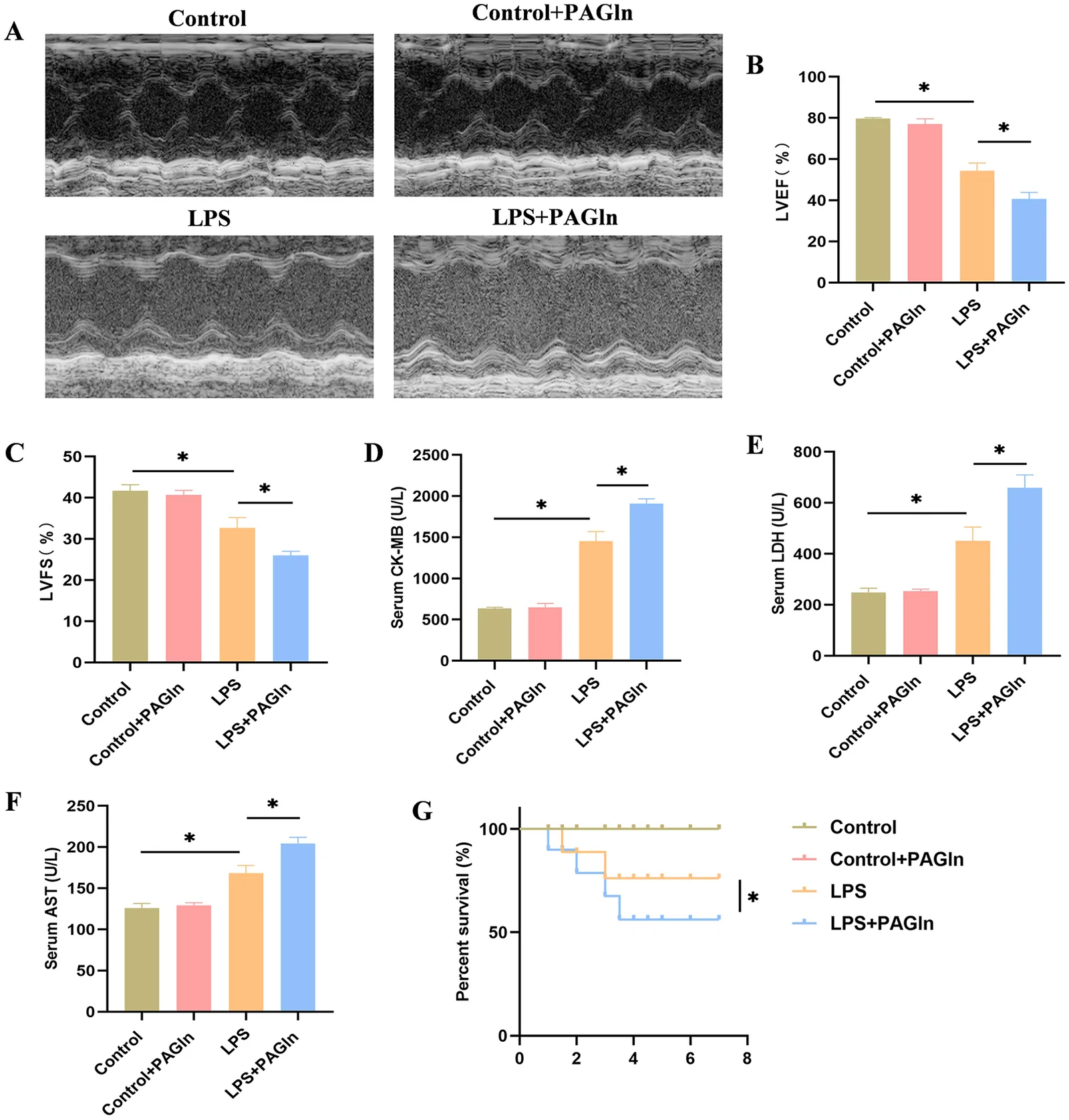
PAGln exacerbates LPS-induced cardiac dysfunction and myocardial injure. **(A–C)** Representative echocardiographic images from each group of mice and statistical analysis of LVEF and LVFS (*n* = 8); **(D–F)** Statistical analysis of serum cardiac injury markers CK-MB, LDH, and AST in mice from each group (*n* = 8). **(G)** Survival analysis of mice in each group (*n* = 10). *P < 0.05. LVEF, left ventricular ejection fraction; LVFS, left ventricular fraction of contraction; CK-MB, creatine kinase-MB; LDH, lactate dehydrogenase; AST, aspartate transaminase.

First, we examined the effects of PAGln on cardiac function. We found that, compared with the control group, LPS-treated mice exhibited reduced left ventricular ejection fraction (LVEF) and left ventricular fraction of contraction (LVFS). Following PAGln administration, cardiac function in LPS-treated mice deteriorated further, as evidenced by a greater decline in LVEF and LVFS (Figures 1A–C). Furthermore, PAGln exacerbated LPS-induced cardiac injury, as evidenced by elevated serum levels of creatine kinase-MB (CK-MB), lactate dehydrogenase (LDH), and aspartate transaminase (AST) (Figures 1D–F). Finally, we analyzed the survival rates of mice in each group and found that, compared with the control group, the mortality rate was significantly higher in the LPS group, and administration of PAGln further increased the mortality rate in the LPS group (Figure 1G). In summary, PAGln exacerbates LPS-induced cardiac dysfunction and myocardial injury.

### 3.2 PAGln exacerbates LPS-induced cardiac inflammation in mice

**Figure 2:**
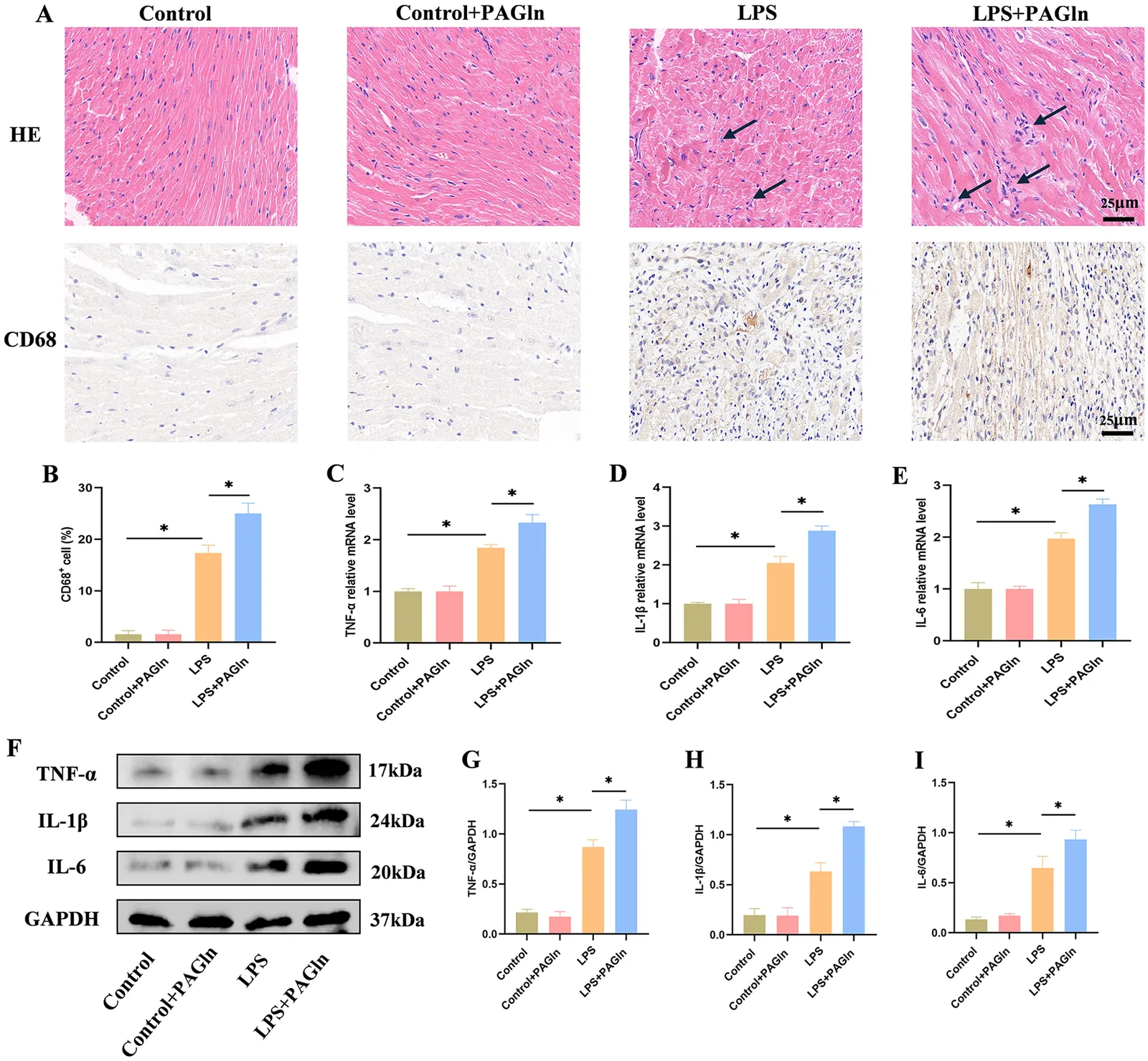
PAGln exacerbates LPS-induced cardiac inflammation. **(A)** Representative images of HE staining and CD68 immunohistochemical staining in each group of mice (*n* = 6); **(B)** Statistical analysis of the CD68-positive area (*n* = 6); **(C–E)** Statistical analysis of mRNA expression levels of the inflammatory cytokines TNF-α, IL-1β, and IL-6 in ventricular tissue from mice in each group (*n* = 4); **(F–I)** Representative Western blot images and statistical analysis of TNF-α, IL-1β, and IL-6 in ventricular tissue from mice in each group (*n* = 3). *P < 0.05. TNF-α, tumor necrosis factor-α; IL-1β, interleukin-1β; IL-6, interleukin-6.

Inflammation represents a key pathological mechanism underlying LPS-induced cardiac dysfunction. Thus, we evaluated the effects of PAGln on LPS-induced cardiac inflammation. First, using HE staining, we found a significant increase in inflammatory cell infiltration in the LPS group, and administration of PAGln exacerbated this infiltration (Figure 2A). Immunohistochemical staining revealed that, compared with the LPS group, PAGln treatment significantly increased the expression of CD68 on macrophages in mice (Figures 2A,B). Furthermore, we found that, compared with LPS-treated mice, PAGln administration led to significantly elevated mRNA expression levels of TNF-α, IL-6, and IL-1β in the cardiac tissue of LPS-treated mice (Figures 2C–E). Western blotting further revealed that PAGln treatment resulted in significantly elevated cardiac protein levels of TNF-α, IL-6, and IL-1β compared to LPS-treated mice (Figures 2F–I).

### 3.3 Effects of PAGln on susceptibility to ventricular arrhythmias in LPS-induced mice

**Figure 3:**
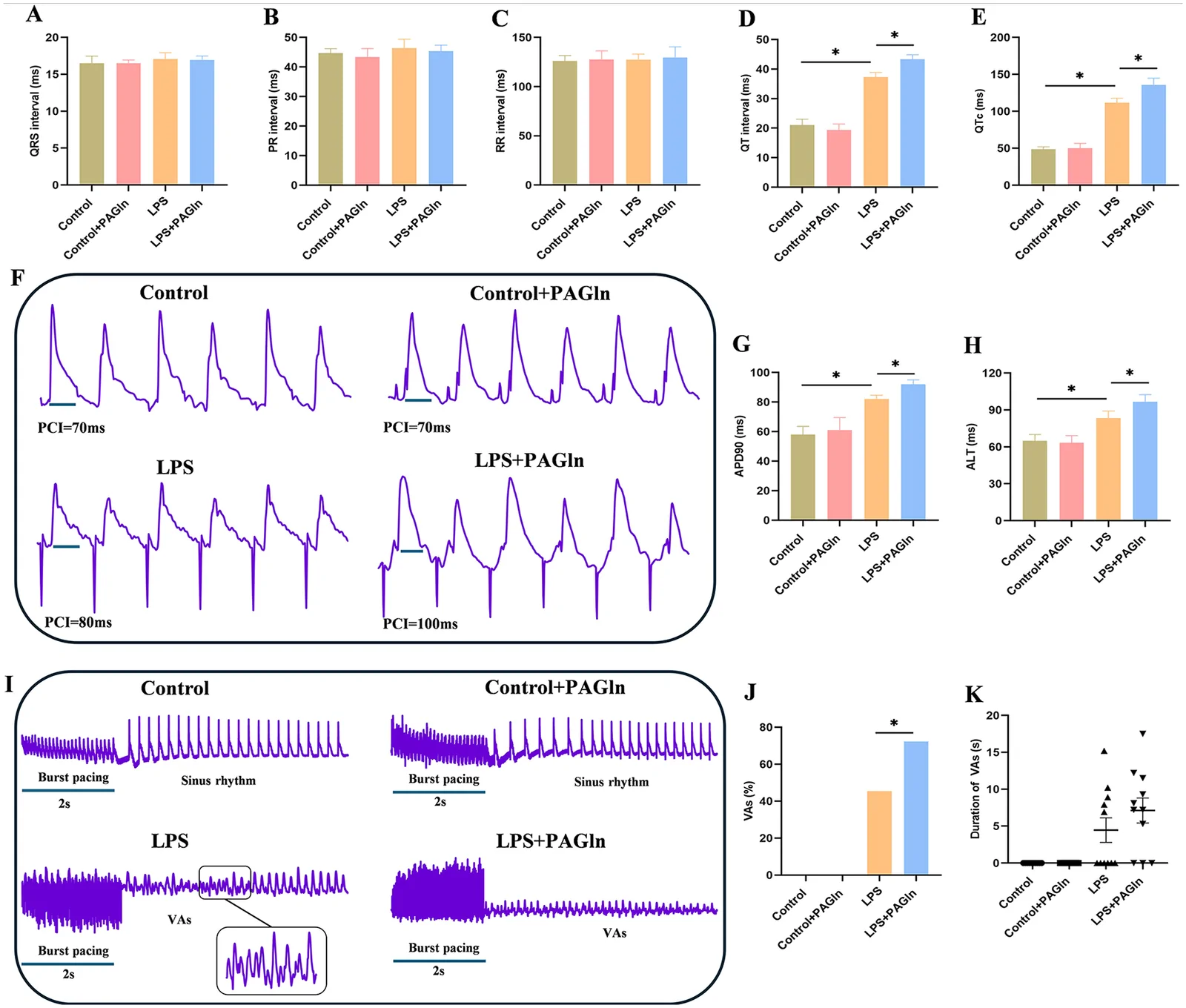
PAGln exacerbates ventricular electrical remodeling and increases susceptibility to VAs in LPS-induced mice. **(A–E)** Statistical analysis of ECG parameters (QRS, PR, RR, QT, and QTc) in mice from each group (*n* = 8); **(F–H)** Statistical analysis of APD90 for mice in each group and representative images of ALT in mice from each group, along with statistical analysis of ALT thresholds (*n* = 8); **(I)** Representative images of burst stimulation in mice from each group; **(J,K)** Statistical analysis of VAs induction rates and durations in mice from each group (*n* = 10-11). *P < 0.05. APD90, 90% repolarization time of action potential duration; ALT, APD alternans threshold; Vas, ventricular arrhythmias.

To evaluate the effects of PAGln on cardiac electrical remodeling in mice with sepsis, we first analyzed electrocardiographic parameters across groups. No significant differences were observed in RR intervals, QRS complexes, or PR intervals among groups (Figures 3A–C). LPS significantly prolonged the QT and QTc interval, and PAGln treatment further increased the QT and QTc interval in LPS-treated mice (Figures 3D,E). Subsequently, we employed in cardiac electrophysiology to investigate the effects of PAGln on ventricular APD, ALT, and VAs susceptibility in LPS-treated mice. We found that compared to the control group, the APD90 and ALT threshold were significantly increased in the LPS group, and PAGln administration further increased the APD90 and ALT threshold in LPS mice (Figures 3F–H). We also observed a marked increase in VAs induction rate in the LPS group, and PAGln administration significantly increased the induction rate of VAs, although there was no significant difference in arrhythmia duration between the LPS and LPS + PAGln groups (Figures 3I–K).

### 3.4 Effects of PAGln on LPS-induced ventricular gap junction protein remodeling in mice

**Figure 4:**
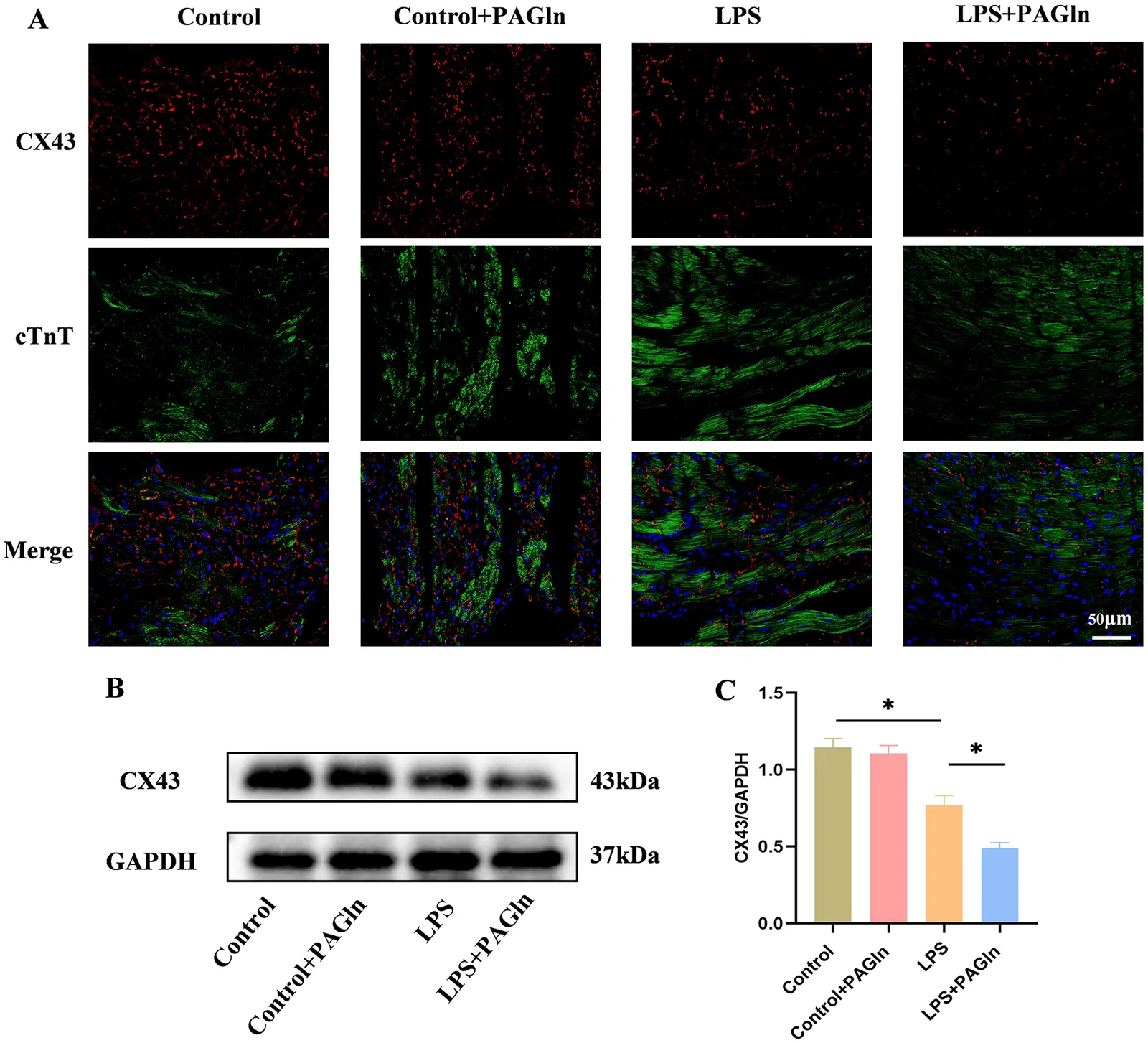
PAGln exacerbates gap junction protein remodeling in LPS-induced mice. **(A)** Representative CX43 immunofluorescence images from each group of mice (*n* = 6); **(B,C)** Representative CX43 Western blot images and statistical analysis for each group of mice (*n* = 3). *P < 0.05. CX43, Connexin 43; cTnT, cardiac troponin T.

Abnormal expression of gap junction proteins in cardiomyocytes is a major cause of abnormal electrical conduction and an increased risk of arrhythmias. Therefore, we evaluated the effect of PAGln on Connexin 43(CX43) expression in cardiomyocytes. Immunofluorescence staining revealed that LPS-treated mice myocardium exhibited significantly reduced CX43 expression, and administration of PAGln further decreased CX43 expression (Figures 4A–C). These findings were consistent with the results of CX43 protein expression.

### 3.5 PAGln exacerbates ferroptosis in LPS-induced mice

**Figure 5:**
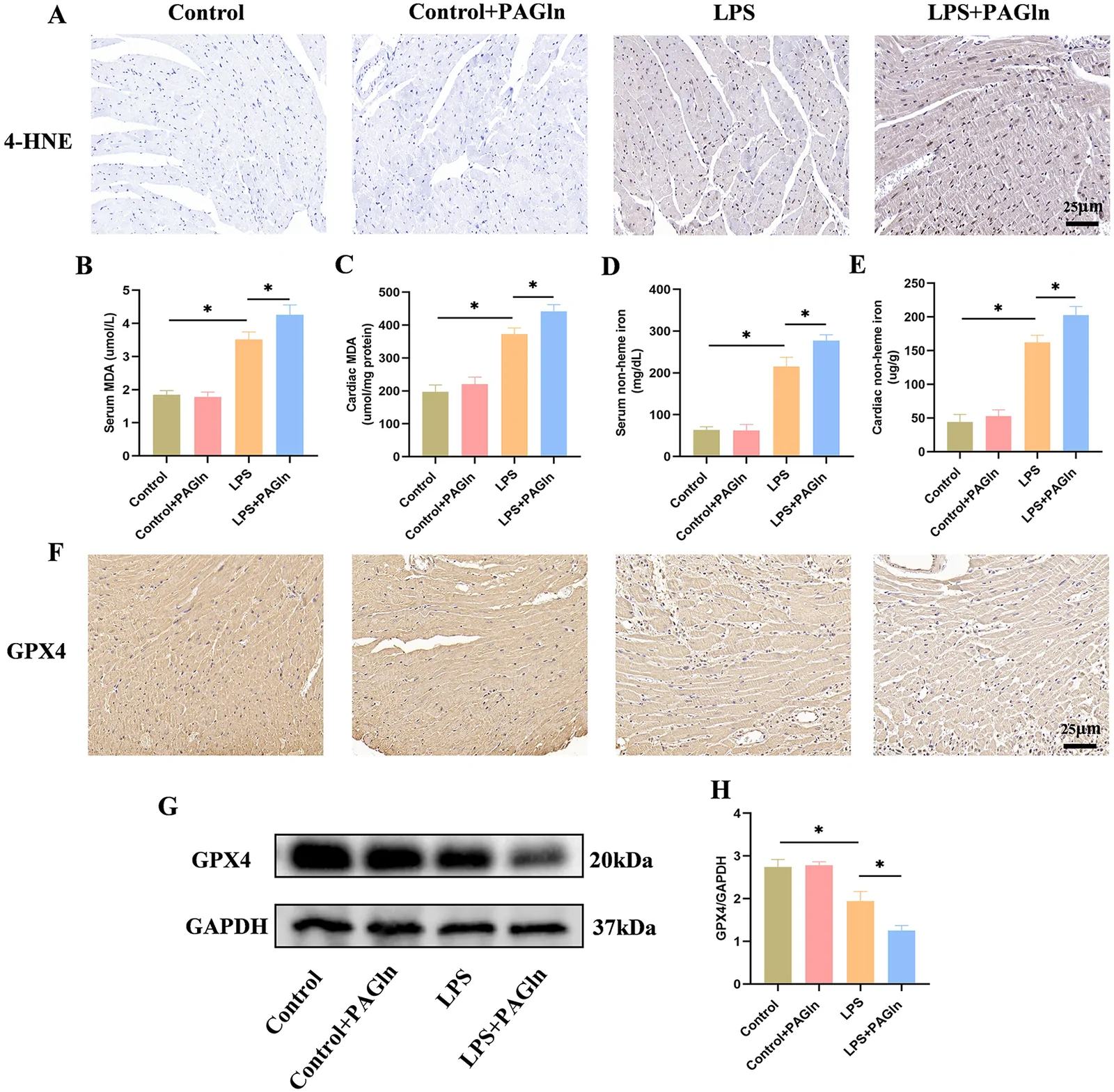
PAGln exacerbates ferroptosis in LPS-induced mice. **(A)** Representative images of 4-HNE immunohistochemistry in mice from each group (*n* = 6); **(B–E)** Statistical analysis of MDA and non-heme iron levels in serum and heart tissue from mice in each group (*n* = 6); **(F)** Representative images of GPX4 immunohistochemistry in mice from each group (*n* = 6); **(G,H)** Representative GPX4 Western blot images and statistical analysis (*n* = 3). *P < 0.05. 4-HNE, 4-hydroxy-2-nonenal; MDA, Malondialdehyde; GPX4, glutathione peroxidase 4.

Ferroptosis is an iron-dependent form of regulated cell death characterized by increased lipid reactive oxygen species, lipid peroxidation, and cell membrane damage. Previous studies have reported that ferroptosis plays a significant role in septic cardiomyopathy (20). Immunohistochemical staining with 4-Hydroxy-2-nonenal (4-HNE) revealed that the degree of lipid peroxidation in the hearts of mice in the LPS group was significantly increased, while administration of PAGln further elevated lipid peroxidation levels in the ventricles of these mice (Figure 5A). Malondialdehyde (MDA) levels in mice serum and hearts were consistent with the histopathological findings, with PAGln exacerbating MDA levels in both the hearts and serum of LPS-treated mice (Figures 5B,C). Furthermore, we found that, compared with the control group, serum and cardiac non-heme iron levels were significantly elevated in LPS-treated mice, and PAGln treatment further increased these levels (Figures 5D,E). Finally, we further examined the expression of ferroptosis-specific markers. Through immunohistochemical and western blot analyses, we found that, compared with the control group, GPX4 protein expression in the ventricles of LPS-treated mice was significantly reduced, and PAGln treatment led to a further decrease in GPX4 expression (Figures 5F–H). These results indicate that PAGln exacerbates ferroptosis in the hearts of LPS-treated mice.

### 3.6 PAGln promotes activation of the TLR4/NF-κB signaling pathway in the hearts of LPS-treated mice

**Figure 6:**
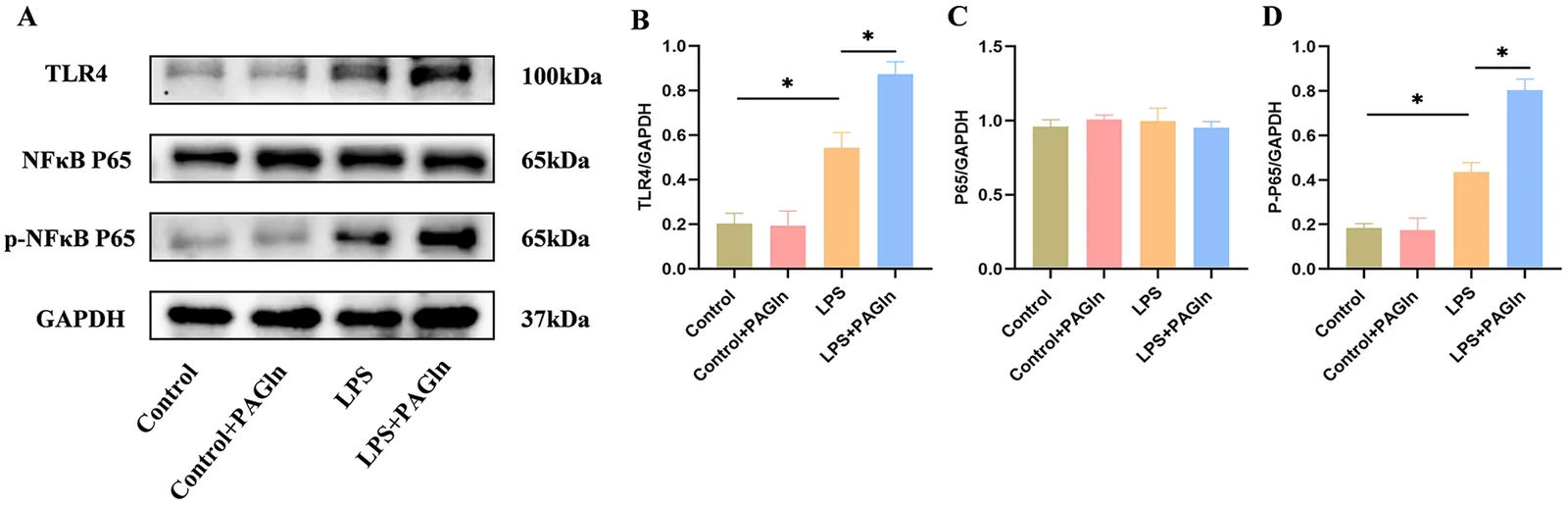
Effects of PAGln on the TLR4/NF-κB signaling pathway in the hearts of LPS-treated mice. **(A)** Representative TLR4, P65, and *P*-P65 Western blot images and **(B–D)** statistical analyses for each group of mice (*n* = 3). *P < 0.05. TLR4, toll-like receptor 4; NF-κB, nuclear factor kappa-B.

Previous studies have demonstrated that the TLR4/NF-κB signaling pathway plays a crucial role in LPS-induced inflammation, which is associated with sepsis-induced myocardial dysfunction (21). Therefore, we investigated the effects of PAGln on the TLR4/NF-κB signaling pathway. Compared with the control group, LPS treatment significantly increased the protein expression levels of TLR4 and *p*-NF-κB p65 in hearts of mice. PAGln administration further elevated the protein expression levels of TLR4 and *p*-NF-κB p65 in LPS-treated mice (Figures 6A–D). These findings suggest that PAGln may promote the activation of the TLR4/NF-κB signaling pathway in the hearts of LPS-treated mice.

## 4 Discussion

In this study, we found that PAGln exacerbates cardiac dysfunction and increases susceptibility to VAs in LPS-induced septic mice. Further investigations revealed that PAGln aggravates LPS-induced cardiac inflammation, potentially through excessive activation of the ferroptosis and TLR4/NF-κB signaling pathway.

Sepsis is a condition typically characterized by a dysregulated host response to infection, which can lead to life-threatening multi-organ dysfunction with high mortality rates and poor prognosis. Cardiac dysfunction is a significant component of multi-organ failure (1). Patients with sepsis who have cardiac dysfunction exhibit a higher risk of death, a finding consistent with the results of our study. In this study, LPS-treated mice exhibited impaired cardiac function, elevated markers of myocardial injury, and a significantly increased mortality rate. The gut microbiota metabolite PAGln further exacerbates LPS-induced cardiac dysfunction and myocardial injury in mice and is associated with a higher mortality rate.

Sepsis can trigger a strong and sustained inflammatory response in myocardial tissue, disrupting the cardiac immune and functional homeostasis (22). The abnormal activation and cascading amplification of inflammatory signaling pathways further exacerbate pathological damage to myocardial tissue, leading to a significant decline in cardiac systolic and diastolic function, and ultimately contributing to the development and progression of sepsis-associated cardiac dysfunction (23). In this study, levels of the inflammatory cytokines IL-1β, IL-6, and TNF-α were elevated in septic mice. The gut microbiota metabolite PAGln has been shown in multiple disease models to be closely associated with either excessive activation or inhibition of inflammation. PAGln can attenuate neuroinflammation induced by intracerebral hemorrhage in mice (24). Furthermore, studies have shown that PAGln promotes cardiac inflammatory responses in mice with heart failure (25). However, in the hearts of septic mice, we found that PAGln exacerbates cardiac inflammation. The anti-inflammatory or pro-inflammatory effects of PAGln may be disease-model-dependent.

Severe sepsis leads to a significant increase in susceptibility to VAs, which are major markers of poor prognosis in high-risk populations (26). Sepsis-induced cardiac electrophysiological remodeling is considered a key factor in the development of malignant arrhythmias (19). Current evidence suggests that prolongation of the QT interval and QTc interval constitutes an arrhythmogenic state and is significantly associated with an increased risk of VAs and mortality (27). Furthermore, electrophysiological abnormalities characterized by ventricular electrical activity—such as prolonged APD and elevated ALT thresholds—are significant indicators of increased susceptibility to VAs (28). Previous studies have shown that PAGln may affect cardiac electrical remodeling. In this study, we also observed prolonged QTc intervals and APD90 as well as increased action potential thresholds in sepsis-induced mice. PAGln affects cardiac electrical remodeling in sepsis-induced mice, thereby increasing susceptibility to VAs.

As a key gap junction protein in ventricular myocytes, CX43 is responsible for maintaining coordinated electrical activity and synchronized contraction in the heart (29). Loss of CX43 leads to abnormal electrical impulse propagation and induces arrhythmias and sudden death (30). In our study, we observed a significant reduction in CX43 expression in the hearts of sepsis-induced mice, and administration of PAGln further decreased CX43 protein expression in the ventricles of these mice, which is closely associated with increased susceptibility to VAs.

Ferroptosis has emerged as a key driver of organ dysfunction in sepsis. Ferroptosis is characterized by iron overload and lipid peroxidation, leading to excessive oxidative damage and cell death. Furthermore, studies have reported that the activation of ferroptosis is closely associated with the occurrence of arrhythmias (31, 32). Our study demonstrates that ferroptosis is significantly activated in cardiac tissue from sepsis-induced mice, manifested by elevated MDA levels, increased iron accumulation, and heightened lipid peroxidation. PAGln significantly exacerbates these features, leading to further accumulation of MDA and iron levels and increased cardiac lipid peroxidation, which is closely associated with increased susceptibility to VAs in sepsis-induced mice. Given the ability of PAGln to regulate iron metabolism and lipid peroxidation, PAGln provides a potential therapeutic target for protecting the heart against sepsis-induced ferroptosis damage and increased susceptibility to VAs.

Accumulating evidence indicates that the TLR4/NF-κB signaling pathway plays a crucial role in sepsis-induced inflammatory responses (33). As one of the innate immune pattern recognition receptors, TLR4 serves a key function in LPS-triggered innate immune responses and initiates inflammatory cascades (34, 35). Furthermore, NF-κB, a pivotal downstream transcriptional effector of TLR4, is also involved in TLR4-mediated inflammation. Activation of TLR4 triggers the activation of NF-κB, ultimately leading to the transcription and massive release of proinflammatory cytokines (33, 36). Previous studies have demonstrated that LPS-induced activation of the TLR4/NF-κB pathway contributes to myocardial injury and arrhythmias (37). Consistent with these reports, our present study also revealed marked activation of the TLR4/NF-κB pathway in the hearts of septic mice. Moreover, the gut microbial metabolite PAGln further hyperactivated this pathway, which was closely associated with excessive inflammatory responses and increased susceptibility to VAs.

## 5 Conclusion

PAGln exacerbate LPS-induced cardiac dysfunction, inflammation, and susceptibility to ventricular arrhythmias by activating the ferroptosis and TLR4/NF-κB pathway.

### 5.1 Limitations

This study has several limitations. First, we have not yet identified the microbial communities responsible for PAGln production in the mouse sepsis model, nor have we characterized their distribution patterns. Second, this study only evaluated the effects of PAGln pretreatment on mice with sepsis; however, the effects of different administration times and doses of PAGln on septic mice remain unclear; Finally, exploring methods to target and regulate PAGln, as well as investigating the association between PAGln and sepsis and clinical outcomes, will help elucidate its value as a novel diagnostic or prognostic biomarker and accelerate the translation of basic research into clinical applications. These issues will be further explored in our future research.

## Data Availability

The raw data supporting the conclusions of this article will be made available by the authors, without undue reservation.
